# A roadmap for safe, regulation-compliant Living Labs for AI and digital health development

**DOI:** 10.1126/sciadv.adv7719

**Published:** 2025-05-14

**Authors:** Stephen Gilbert, Rebecca Mathias, Anett Schönfelder, Magdalena Wekenborg, Julia Steinigen-Fuchs, Anja Dillenseger, Tjalf Ziemssen

**Affiliations:** ^1^ Else Kröner Fresenius Center for Digital Health, TUD Dresden University of Technology, Fetscherstr. 74, 01307 Dresden, Germany.; ^2^ Member of the ethics committee at TUD Dresden University of Technology, Fetscherstr. 74, 01307 Dresden, Germany.; ^3^ Center of Clinical Neuroscience, Department of Neurology, University Hospital Carl Gustav Carus, TUD Dresden University of Technology, Fetscherstr. 74, 01307 Dresden, Germany.

## Abstract

Safe and agile experimentation spaces are essential for developing AI-enabled medical devices and digital health innovations. “Living Labs” offer a collaborative environment for testing technologies in near-real-world settings, capturing user perspectives and outcomes, often missed in traditional testing. However, the flexibility of Living Labs often clashes with the European Union’s rigid regulatory frameworks for medical devices that were not designed for digital technologies. We examine this intersection, showing how flexibility in evaluation and patient interaction can coexist with safety guardrails. Our approach integrates technology readiness, preemptive planning, and iterative modifications to bridge innovation and regulation, fostering adaptive health care technology development.

## Teaser

A Living Lab framework can support compliant development and evaluation of AI-enabled devices suited to clinical workflows.

## INTRODUCTION

The rapid growth of artificial intelligence (AI)–enabled technologies holds the potential to address critical gaps and improve health care (*1*, *2*), yet it also raises valid safety concerns regarding their adoption and use (*3*–*5*). As innovation and digitalization in the medical device sector accelerate—particularly in the areas of digital patient and health care provider (HCP) apps, artificial intelligence, and wearable sensors (*6*, *7*)—aligning these advancements with suitably adapted regulatory frameworks that ensure safety while not overrestricting flexibility has become increasingly important (*8*–*13*).

Unlike traditional medical devices (MDs), AI-enabled digital health technologies (DHTs) are trained on data and can be locally, periodically, or continuously adapted during use. Similarly, non–AI-enabled DHTs often undergo adaptations based on user feedback through agile software development approaches (*5**,* *10**,* *11*). International guidelines (*14*–*18*) stress the importance of training AI devices on datasets that represent the patient population and clinical environment while considering the total product life cycle, especially for adaptive systems. However, current frameworks treat DHTs as stand-alone entities, whereas modern digital devices are increasingly flexible, connected, and interdependent (*8*, *9*). Although regulatory frameworks are beginning to recognize that these technologies should be adaptable on the market (*10*, *11*, *14*, *16*, *17*, *19*), many aspects remain suitable only for static devices.

Globally, the need for a shift in regulatory thinking varies, with some authorities like the US Food and Drug Administration (FDA) being more open to acknowledging that current regulatory frameworks, although well intentioned, lead to narrow siloed DHT development, where developers are neither able nor incentivized to adopt system-level holistic thinking (*20*). This is evident in the FDA’s regulatory science efforts to explore flexible suites of devices (*8*, *9*, *21*) such as the recently launched Health at Home Hub Initiative (*20*, *22*). Similarly, Singapore has taken a proactive stance with its Smart Nation program, which has driven several AI-focused initiatives, including the adoption of a National AI Strategy in 2019, later renewed in 2023 (*23*). While programs like these lay the foundation for innovation in AI-driven health care, it will take time before such approaches translate into new rules or guidance and are adopted on a large scale across health care systems.

The current approach in regulatory frameworks is to ensure the safety of DHTs (and other medical devices) by requiring developers to consider, understand, and manage all potential interactions with other devices (*8*, *9*). However, these regulations often impose a substantial administrative burden on the developer, limiting the exploration of flexible, real-world scenarios. This restricts the integration of data from participatory testing and user feedback during development, including complex interactions with patients, health care professionals, and other technologies (*24*). In practice, usability evaluations are often confined to isolated research activities disconnected from real care delivery, offering limited insights. When studies extend to assessing actual care delivery, they often cross the threshold into clinical investigations (i.e., clinical trials), which come with heavy administrative demands and rigid protocols that allow little flexibility (*25*–*27*), and thus discourage optimal experimentation that could uncover real-world challenges and interoperability issues. Given the administrative burden associated with applying for permission to conduct flexible explorations, developers are effectively pushed to limit their focus. This results in a situation where they do not consistently evaluate factors like integration into clinical workflows and user-friendliness, nor do they often foster support for the testing of these parameters in truly interactive circumstances during development (*20*, *24*). As a result, interoperability and challenges associated with system-level interactions are often only detected after the DHT is placed on the market, if at all.

**Fig. 1.**
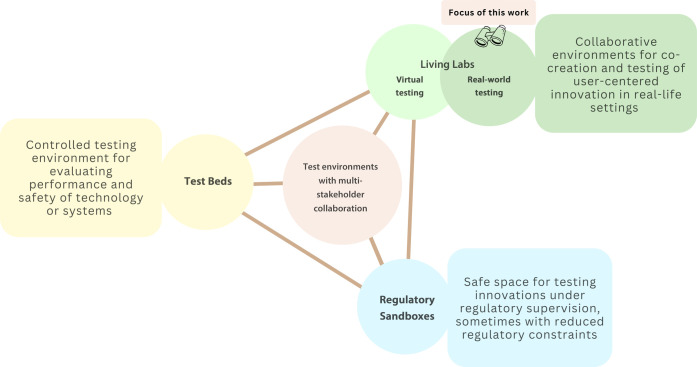
Current testing and experimentation environments that involve multistakeholder collaboration. Relevant to DHT development are Test Beds, Living Labs, and Regulatory Sandboxes. Here, we focus on Living Labs involving real patient and HCP interactions in a real-world clinical environment, enabling risk-controlled experimentation. This figure was created using Canva Pro.

Options that have been proposed for addressing these challenges include experimental environments like Living Labs, Regulatory Sandboxes, and Test Beds (*28*) (Fig. 1). Living Labs are environments designed to generate evidence and test innovations that are challenging to explore within restrictive traditional clinical study parameters. Rather than replacing clinical trials, Living Labs adapt the rigorously managed trial environment into a more flexible, real-world setting, allowing safe exploration of varied uses and interactions across development stages. This approach fosters a cocreative space for testing innovative methods and examining technology interactions, which could enable easier transfer into practice.

The benefits of Living Labs have been highlighted in previous research, but conclusive evidence remains limited, possibly due to concerns regarding the feasibility of their implementation, their regulatory oversight, and costs related to their setup. Some results have been promising, such as a 2019 publication based on survey data from 92 Living Labs across multiple sectors and nations, which showed that user-centered and participatory approaches improved performance outcomes—reducing costs, accelerating time to market, and enhancing quality (*29*). Another literature review including 30 studies found that the Living Lab concept could improve the acceptability and feasibility of implementing emerging health care innovations in real-world settings (*30*). As the concept of Living Labs is not exclusive to the health care sector, insights from other fields also offer valuable lessons. For example, a study using SWOT analysis on the use of Living Labs in Sweden’s building sector demonstrates that Living Labs can accelerate innovation in sustainable building technologies through real-world testing, fostering industry-academic collaboration, and providing a financially sustainable, scalable model for cocreation and research in smart buildings (*31*).

This paper explores the regulatory and ethical requirements, as well as challenges to establishing and effectively using Living Labs in health care, that involve real patients in real treatment scenarios, with a focus on the European Union (EU). We discuss the degree to which current regulations facilitate or hinder the use of Living Labs and suggest ways to navigate these frameworks while maximizing the benefits for product development and ensuring that sound patient safety guardrails are always in place. Although EU-focused, we emphasize the importance of ethical safeguards and gathering meaningful data that can be fully used while maintaining the highest privacy standards. Our suggestions are applicable globally, as they are based on universal ethical principles rather than specific national regulatory requirements.

Building on our own experience of establishing a university hospital–based Living Lab for Multiple Sclerosis research, we set out a landscape for Living Labs with a focus on the exploration of DHTs in the care pathway and our interactions with all stakeholders, including those responsible for regulatory and ethical oversight (*32*). Our established Living Lab is an evolving concept and is continuously adapting based on interaction with these stakeholders (*33*). In addition, we draw insights from a second Living Lab hosted on our campus as part of the internationally funded Health Labs4Value project, which aims to enhance patient care in Central Europe by facilitating the transfer of digital solutions through collaborative environments (*34*, *35*).

This article focuses on Living Labs in medicine, involving real patients and HCPs interacting with real DHTs in physical environments (Fig. 1). We exclude virtual/simulated or extended reality environments (*20*, *28*, *36*), which currently have limited validity in simulating complex medical scenarios and operate under different regulatory and organizational conditions. Our focus is on Living Labs in university medical facilities without formal involvement of regulatory bodies for regulatory learning or special regulatory flexibility beyond applicable laws (*37*–*42*). We explore Living Labs to evaluate DHTs, including software as a medical device (SaMD) and AI-enabled SaMD, and the interaction of software with sensor and wearable technologies. Living Labs can be used to advance DHTs through different stages of development according to their technology readiness levels (TRLs) between TRL 3 (proof of concept) and TRL 7 (approved prototype) (*43*). The TRL (*43*) development scenarios considered are the following: (i) fully internal development by the clinical center of a proof of concept (TRL 3), not intended for use in patients outside carefully controlled risk scenarios in a clinical investigation; (ii) the transition of a proof of concept, through clinical validation in the clinical use environment, leading to ongoing use within the clinic (interface of TRL 6 to TRL 8); (iii) further advancing such a concept to regulatory approval and placing a product on the market (TRL 9) through the involvement of a spin-off company or an external enterprise or by supporting an external enterprise in obtaining such approval, providing postmarket clinical data, or extending their approval for an on-market product.

## LIVING LAB APPROACHES IN MEDICINE

The term “Living Lab” has no formal definition and was introduced in the early 2000s in Europe as a description for real-world environments to test disruptive technologies, and has since been adopted in health care (*30*). By 2024, the European Network of Living Labs (*44*) registered more than 480 members worldwide across various disciplines. Typically, Living Labs emphasize user-centricity, cocreation, and real-world testing, seeking to develop usable products with input from diverse stakeholders (*30*). Some of these approaches have been based on the Quadruple Helix Model of innovation, where government, academia, industry, and society collaborate to support technology development from conception to market (*44*).

The implementation of Living Labs differs widely because of factors like location, stakeholders, funding, and objectives (*45*–*47*). The regulatory and ethical considerations for their operation vary on the basis of the nature of the Living Lab, which depends on the specific goals of those setting them up and managing them. They can explore any stage of development across the TRL scale, from TRL 1 to TRL 9 (*28*, *43*).

## TENSION BETWEEN LIVING LABS AND REGULATORY FRAMEWORKS

The primary purpose of Living Labs is to provide a flexible environment to cocreate, prototype, test, and upscale innovations in the setting of uncontrolled (near) real-world environments (*28*). This concept relies on flexibility and the ability to adapt the following in near real time: (i) use scenarios, (ii) features of device design, (iii) the interactions between devices and humans (i.e., patients and HCP) and their workflows, and (iv) measurement and test scenarios (*28*, *30*, *48*–*50*).

**Fig. 2.**
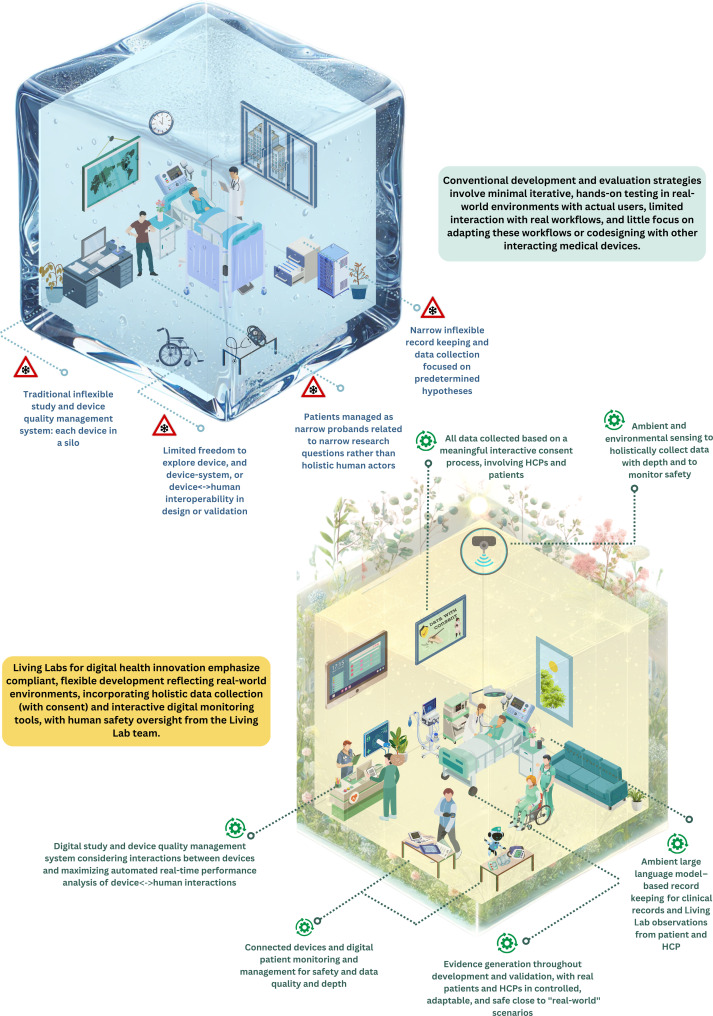
Comparison of the conventional development of DHT and the Living Lab approach. The upper panel depicts conventional evaluation strategies that use minimal experimentation within the regulatory framework, resulting in minimal flexibility in further or participatory development or in testing strategies. The lower panel depicts how a Living Lab can enable flexible and safe codevelopment and evaluation of technologies and can stretch traditional regulatory boundaries while maintaining patient safety by leveraging digital patient, HCP, and environmental monitoring. This figure is a representation of a Living Lab, which can be adapted to meet actual needs. It was created by-hand by the authors, with all conceptualization and design created through human effort, and AI tools were used only for minor visual enhancements to specific components. Figure credit: Concept authors, R.M. and S.G.; figure creation author, R.M. This figure was created using Canva Pro.

Medicine allows a wide range of flexibility in the use of innovative approaches, provided that they align with the state of the art, adhere to national and international medical guidelines, and remain within the scope of the Hippocratic oath (*51*). When innovation in practice crosses over into the testing of approaches, the Declaration of Helsinki (*52*) and Good Clinical Practice apply (*53*, *54*). These frameworks mandate detailed protocols, informed consent, and verification of site and principal investigator suitability, ensuring that risks are estimated and managed through clear study plans specifying what will be tested and how. Medical device (including AI-enabled devices) regulation is even more prescriptive, requiring that the device must be designed and tested for a specific purpose (*39*, *42*, *53*). This creates a natural conflict between the flexibility inherent to Living Labs and the predetermined approaches mandated by regulatory and ethical frameworks (Fig. 2).

## RIGID NATURE OF EXISTING FRAMEWORKS

Medical device development pathways are often viewed as rigid, making the concept of Living Labs seem unrealistic. To better understand this, it is important to explore the regulatory context, identifying where inflexibility is explicitly enforced, where it arises as a secondary consequence of other regulatory requirements, and where overly strict interpretations of regulations can restrict freedom in the development and clinical validation of devices, potentially limiting innovation.

### Evaluating the feasibility of new DHT concepts (TRL 1 to TRL 3)

Experimental studies typically begin at TRL 3, where a high-level concept (in this case, related to software or AI) is explored. If the goal is to advance the scientific literature, and the product does not qualify as a medical device by definition, nor is there any intention to develop one, it is unlikely that the study will be considered a medical device study [by definition in Articles 62 and 82 of the Medical Device Regulation (MDR)] (*39*) by either the ethics committee or regulatory authorities. However, because these studies involve direct use of technologies and research with patients and health care professionals, ethical approval is still required, and this will require the submission of a detailed and specific study protocol. In Germany, studies of this type would generally be approved under the Medical Professional Code of Conduct (“Berufsordnung” studies), and both the tool investigated and the protocol for the study are fixed. Any changes to these require a formal amendment of the study protocol, followed by reapproval by the ethics committee. Some researchers may feel tempted to approach the early development of a medical device under this means, describing it as a study of a research concept when they are intending to develop and validate a medical device. This is an inappropriate approach as it contradicts legal and ethical frameworks designed to protect patients and leads to wasted efforts, as the data collected in such studies are often unusable (generally neither the device nor study is adequately documented, and the data have little utility for approval processes).

### Early clinical validation of DHTs (TRL 4 to TRL 6)

For TRL 4 to TRL 6, it is acknowledged that the tool under investigation is an in-development medical device. Regulatory frameworks, guidelines, and standards mandate detailed technical documentation outlining its development based on user needs and compliance with applicable regulations (*39*). A comprehensive testing plan must also be established to demonstrate that all requirements are met, shaped by ongoing risk assessments, and include a predefined strategy for clinical evaluation with acceptance criteria for clinical data. Clinical investigations conducted at different stages of development—early-stage studies (MDR Article 82) and approval-focused studies (MDR Article 62)—must adhere to the detailed technical documentation and investigation protocols (*39*). These studies require notification or approval from the competent regulatory authority (e.g., in Germany, Article 82 investigations require notification and Article 62 studies require approval of the competent authority). Protocol amendments must be reviewed and approved by the relevant authorities and ethics committees to ensure compliance.

### Late clinical validation of DHTs (TRL 7 to TRL 9)

For TRL 7 to TRL 9, the requirements for later-stage validation of a medical device are similar to those described above for TRL 4 to TRL 6 but with greater expectations that the device and study protocols are fixed (*39*, *53*). Clinical investigations conducted to generate clinical data for market approval are regulated under MDR Article 62 and require extensive documentation of the study procedure. Requirements for postmarket studies are similar (MDR Article 74), with some minor exceptions to requirements, for example, in many EU Member States, patient consent is not required if there is no study intervention and no burdensome procedures (*53*).

## MAXIMIZING LIVING LAB POTENTIAL UNDER CURRENT FRAMEWORKS

The need for highly developed documentation for devices and highly predetermined protocols, as described above, appears to stifle the flexibility that is inherent to the Living Lab experimentation concept. However, the use of environmental and patient monitoring technologies alongside the automation of quality processes (*55*) is beginning to enable the rethinking of evidence generation. Regulatory frameworks are already evolving to formally and directly recognize the need for experimentation spaces [e.g., predetermined change control plans (PCCPs) for adaptive AI DHTs (*16*, *56*) and Regulatory Sandboxes under the AI Act (*41*, *57*)]. Over time, there will be further evolution of the regulatory frameworks to enable Living Labs. We set out a framework for Living Labs compatible with current regulation, but local implementation will require early engagement with the responsible ethics committees, competent authorities, hospital structures, and, where relevant, Notified Bodies.

### Automated quality control, safety, and ethics monitoring

Living Lab flexibility must operate without compromising integrity or bypassing safety frameworks. Thus, in certain aspects, Living Labs in medical device development must be highly controlled and observed. It is this high level of control and observation that enables meaningful freedom. An “anything goes” approach is not applicable to medical Living Labs. Where human patients and HCPs interact with evolving technologies, compliance with the Declaration of Helsinki (*52*) remains nonnegotiable. Successful implementation requires careful planning, a dedicated strategy, a multistakeholder operations and quality team, strong communication (*58*), and adequate resources.

While balancing greater accountability and freedom may seem paradoxical, advances in ambient/contact-free and wearable AI-enabled devices that were not available before now bring this within reach. The digital nature of devices that use optical sensors or radio frequency to monitor vitals like temperature, blood pressure, and oxygen levels, as well as AI-powered systems that analyze behavior, breathing, and mobility without wearable sensors, can be transformative when used effectively in this setting. These approaches allow comprehensive and continuous data collection that is critical to the observation of the Living Lab and, in turn, critical to the safety of patients and the reliability of recorded data and outcomes. These tools enhance decision-making, personalize treatment, and improve patient compliance while facilitating environmental observation and measurement, with applications already seen in clinical studies (*59*). They enable comprehensive data collection to support device and workflow evaluation, increase real-time observation and intervention to protect patient safety, and ensure HCP compliance (*60*). In addition, efficient data management helps identify errors, streamline regulatory processes, support compliant data sharing, and enhance both patient care and quality management (*61*, *62*).

A potential advantage of these approaches is the ability to observe which aspects of experimentation have remained static and which have changed, offering a unique opportunity to examine variables either individually or in combination. This has the potential to be particularly valuable in Living Labs exploring DHTs where aspects of the same platforms (e.g., a smartwatch) can be used as a host environment of the DHT and as a data recording environment for the Living Lab to study compliance and observe safety. Continuous monitoring during interventions is key to ensuring that patient safety is not compromised while experimenting with these variables. These approaches are not yet used to their full potential with inconsistencies in information and communication technology tools used across various Living Labs (*62*).

When applying AI and digital monitoring in Living Lab setups, it is important to consider ethical dimensions and how to control these to ensure responsible research and development. These include the avoidance of bias, discrimination, lack of transparency, and potential misuse of sensitive data. Living Lab protocols must prioritize adherence to the ethical principles and constitutional protections of their host nation, including fundamental rights such as human dignity and personal freedom. Within the EU, it is essential to ensure compliance with legislation such as General Data Protection Regulation (*63*). Similarly, other legislations such as the recently adopted AI Act (*41*) apply, with certain provisions that came into effect on 2 February 2025, focusing on prohibitions and AI literacy obligations. It is critical that new technologies for contact and noncontact observation are linked to tailored concepts of dynamic, broad, and hybrid consent management that all allow patients (and, in some circumstances, HCPs) to carefully manage in real time what they do and do not allow to be observed and recorded (*64*–*66*). Respecting these foundational rights is essential for fostering trustworthy and ethically sound innovations.

### Living Lab, user, and stakeholder needs (TRL 1 and TRL 2)

The Living Lab concept is particularly well suited for early innovation at TRL 1 and TRL 2, where it fosters an environment for identifying and addressing stakeholder needs, bridging the gap between users, developers, and compliance experts. For successful outcomes, a critical aspect of medical device development, including DHTs, is understanding user requirements and translating them into clear product specifications (*24*). At this stage, technologies are typically applied in an observational or highly derisked manner, leading to a relatively lighter regulatory oversight burden compared to later development stages (*39*, *67*). Conducting this early-stage research in a Living Lab is particularly valuable for gathering rich data on potential interactions between prototype DHTs, patients, HCPs, other devices (including prototypes), and the environment, all within a flexible research setting. Ethical approval for such flexible study designs in Living Labs is determined on a case-by-case basis by the responsible ethics committee. However, the distinction between predevelopment research and the start of regulated device development remains a gray area (*67*). Irrespective of whether the frameworks for formal medical device development are judged to apply, it is essential to have clear standard operating procedures (SOPs) that form the foundations and thresholds of what can and cannot be undertaken. These SOPs must clearly set out the framing conditions for a well-designed environment of research, clearly specific data collection, and documentation processes. If so, they improve the likelihood of approval by the ethics committee, maximize the efficacy of developing concepts through successive TRL stages, and optimize the use of data. Although a framework of SOPs could be seen as contradictory to “experimentation spaces,” by adopting brevity, discipline, and intelligence in the design of procedures and ensuring that these procedures are supported by structures, automated systems with human oversight where necessary, and roles working holistically together, the Living Lab can operate in a way that is useful, safe, and ethical.

When humans are involved in research conducted by medical doctors or HCPs, it is typically necessary to consult the responsible ethics committee. Depending on the research, additional consultations with the hospital’s staff council or management board may also be required [e.g., in Germany (*68*)]. The ethics committee may issue a waiver or require a full submission and opinion. In the scenario of TRL 1 and TRL 2 described here, where there is no specific test device (instead, there is a nondevice tool or loose software concept) and the research is primarily qualitative and exploratory, ethics committees are likely to permit relatively flexible approaches, such as adaptive interview designs, provided that the study protocol clearly defines the range and limits of this flexibility.

### Living Lab permissibility for MD studies (TRL 3 to TRL 8)

For TRL 3 to TRL 8, both studies for evaluating high-level scientific concepts (i.e., studies of nonmedical devices) and formal clinical investigations of in-development medical devices require the predevelopment and preassessment of a detailed protocol, which must be considered in detail by ethical oversight bodies (and, for medical device studies, this must also be submitted to regulatory bodies). Although these protocols and their associated risk assessments generally set out narrow research concepts for narrowly defined devices settings, the operators of a Living Lab concept do, at least in theory, have the freedom to propose to the oversight ethical and regulatory bodies a wider concept or “envelope” of evaluation and/or potentially include the in-study feedback-driven iterative modification of the device under evaluation. If such an approach is to have a realistic chance of approval, it is critical that the “adaptable” aspects of the study are well thought out and their bounds thoroughly described and justified within the study plan documents.

A roadmap to a bounded but flexible approach, which sets out thresholds alongside the management of change, can be seen in recent concepts for PCCPs in medical device approval and postmarket adaptation for both AI-enabled and non–AI-enabled agile-developed SaMD (*10**,* *11**,* *16**,* *56*). It is noted that these are envisaged for the postmarket phase of device development and not for early stages, but we argue that the same overall approach is applicable to early development. These approaches enable the regulatory approval of devices with a defined “envelope” or “prespecification” of performance while permitting adaptive changes during the postmarket period (*10*, *11*, *56*). Ethics committees and regulatory bodies are likely to accept proposals based upon adaptable devices in early or later validations studies using the same or similar description of an “envelope” of permitted changes. Specifically, this would consist of a PCCP for the investigational device, allowing for its adaptation between study stages, which defines the scale and nature of the changes in device properties and performance that are allowed without a formal study amendment, as well as those changes that require a specific amendment. It might be possible to arrange fast-track and agile modes of interaction, with ethics and regulatory oversight bodies, for those circumstances where formal protocol amendments do need to be evaluated, thus enabling these processes to be carried out promptly and without disrupting the continued running of the investigation in the Living Lab.

An example of a flexible approach applied in evaluation of DHTs for a TRL 4 and TRL 5 DHT app prototype was explored in a two-phase mixed method pilot evaluation, carried out in a hospital emergency department by Scheder-Bieschin *et al.* (*69*). The first phase of the study included data gathering from structured interviews, itemized questionnaires, and direct observations. This study phase was accompanied by members of the app product team, including the product owner, designer, and user experience researchers, who directly interacted with the principal investigator and study team on-site, including participating HCPs and patients. The phase-one data, as well as patient and user feedback, were not used as part of the formal evaluation of the DHT but instead guided the iterative improvement of the digital tool. Between study phases one and two, further targeted and time-windowed adaptive development and bench testing of the DHT were carried out by the app product development team. The modified app was then further evaluated in phase two, with the results from this stage representing the definitive results of the pilot evaluation of the second iteration of the prototype DHT.

**Fig. 3.**
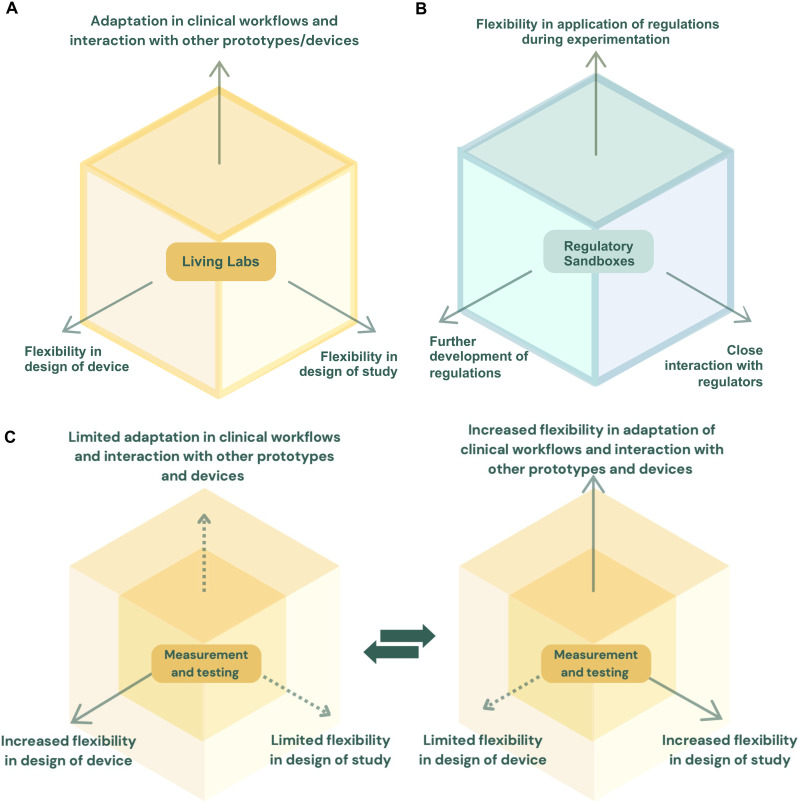
Flexibility of Living Lab environments and their relationship to Regulatory Sandboxes. (**A**) Different degrees of flexibility offered by Living Lab environments. There are three dimensions: (i) flexibility in ongoing development of the DHT; (ii) flexibility in the ongoing development of research data measurement and evaluation methodologies; and (iii) flexibility in interaction of the DHT with other DHTs and health systems and workflows. (**B**) Different degrees of flexibility offered by Regulatory Sandbox environments. There are three dimensions: (i) close interaction with the regulator and sometimes (ii) further development of regulations and (iii) a degree of flexibility in the application of the regulations while in the experimentation spaces. (**C**) Toggling Living Lab flexibility phases. This figure was created using Canva Pro.

This approach brought together two of dimensions of flexibility that can be included in Living Labs: (i) flexibility in study design (i.e., flexibility in measurement), introduced through the use of qualitative user-experience research methods with real users in the real use scenario; and (ii) flexibility in device design (i.e., flexibility of the thing being measured), achieved through the iterative changes in the device during further development and bench testing, concurrent with the study, and only interrupted by a 2-week pause. This study only minimally explored a third dimension (iii), which could allow for simultaneous adaptation of clinical workflows and the exploration of interactions with other prototypes under development, supported by real-time data capture and processing supported for real-time compliance, safety, and ethical safeguarding (Fig. 3A). Depending on the clarity of planning, quality of design, and the transparency maintained with overseers, it might be possible to get an approval for a Living Lab operation that includes both flexibility in research instrument design and the testing of iterative SaMD versions adapted on the basis of early study phase results and feedback. These approaches are not expressly forbidden in the regulations for clinical investigations, but the degree of flexibility described here is unusual in practice. Therefore, an exceptionally high degree of transparency to maintain open communication that builds trust with overseers, care in study design, and use of monitoring will be needed to justify the merits of this flexible approach to ethics committees and regulatory bodies.

### Living Labs and devices for sole in-clinic use (TRL 3 to TRL 8)

For TRL 3 to TRL 8, in the EU, individual health institutions are allowed, under specific conditions, to design, manufacture, modify, and use in-house medical devices on a nonindustrial scale (i.e., those developed and for use within the individual health institution itself). This is allowed under specific conditions if the needs of patient groups cannot be adequately met through available market devices (*39*, *70*). It is a requirement that safety and ethical standards are upheld, and although competent authorities theoretically have an oversight remit, this is much looser than that is applied to generally marketed medical devices, and it does not involve third-party assessment. An implication of this regulatory framework is that it means that, in practice, pseudo–Living Labs already function in hospitals that develop in-house medical tools, particularly when these are DHTs. This has advantages in that it enables the flexible and cocreated real-time development of DHTs within the same institution. In addition, as development and (to practical considerations) oversight are in-house, there can be the flexible integration of multiple DHTs into workflows and into interactive work patterns, with further participative refinement of the technologies, which is often difficult under more formal regulatory requirements for on-market medical device development. According to Medical Device Coordination Group guidelines, combining or modifying devices in-house to form a new device intended purpose still qualifies as in-house manufacturing, (*70*, *71*), provided that these activities remain within the scope of the intended purpose as defined by Article 5(5) of the EU MDR (*39*). A disadvantage of these “in-house manufacturing” rules is that often (in the observation of the authors) adequate safety safeguards in development, quality management, and surveillance principles are not applied. Although a substantial financial and effort investment is needed, we argue that the health institution developers should adopt the processes of quality oversight, suited to flexible Living Labs, that we have described. As well as better ensuring patient safety, the advantage of properly developing in-house DHTs within adequate (but flexibly constituted) quality management systems is that these tools could then readily be migrated to market-approved tools, through the market approval pathways, as development documentation and validation data collected would be suited to approval (i.e., CE marking). Considering in-house development as overlapping, the Living Lab approach we describe would facilitate spreading from in-house to wider health system market-approved “out-of-house” general market use and hence allow well-developed DHTs to deliver population-level health benefits, greater health system economic efficiency, commercial advantages for hospital groups through the licensing of patents and other intellectual properties, and economic benefits through small and medium enterprises further developing these technologies.

### Linkage of Regulatory Sandboxes and Living Labs (TRL 3 to TRL 8)

For TRL 3 to TRL 8, a conceptually adjacent experimentation environment to Living Labs is Regulatory Sandboxes (Fig. 3B). The latter concept brings the developers of DHTs close together with the regulators in a common environment (i.e., the “sandbox”) where there is (i) closer communication with regulators (*41*, *57*), (ii) the coexploration or experimentation spaces for the further development of regulations (*11*, *72*, *73*), and/or even (iii) a certain degree of “playroom” in the application of regulations. These concepts can overlap in sandboxes to differing degrees (*74*–*76*).

Although Living Labs and Regulatory Sandboxes are both experimentation spaces relevant to DHT development, they could be seen as separate domains. However, we argue for the advantages of merging these concepts so that the benefits of both areas can be combined. In this combination, Living Labs bring the three dimensions of flexibility (Fig. 3A): (i) flexibility in the ongoing development of the DHT; (ii) flexibility in the ongoing development of research data measurement and evaluation methodologies; and (iii) flexibility in interaction of the DHT with other DHTs and health systems and workflows. Sandboxes bring (i) close interaction with the regulator and sometimes (ii) further development of regulations and (iii) a degree of flexibility in the application of the regulations while in the experimentation spaces (Fig. 3B).

### Toggling Living Lab flexibility phases

Living Labs offer the opportunity for safe flexible exploration of in-development DHTs, facilitated through innovative approaches to oversight, safety observation, and dynamic monitoring and adaptation of compliance mechanisms (Fig. 3A). How can rigorous establishment of safety be ensured when all three dimensions are constantly in flux? The property of flexibility does not mean that everything should be changed along all dimensions in a Living Lab simultaneously. Under current monitoring and evaluation technologies, this would make no sense. The operators should therefore move the levers of flexibility with care and where needed and might initially explore only one axis of flexibility. In general, changes in the flexibility dimension should not be at the same time, and effects of changes should be well characterized before further changes are made and only within the threshold of change allowed by the regulatory frameworks and the SOPs of the Living Lab. Early in the development of a specific DHT, it makes sense to have relatively high degrees of flexibility in the Living Lab. In later clinical validation stages of a DHT, it is needed to have less flexibility to provide definitive data under tighter controls. There is therefore a need to toggle Living Lab between flexibility phases depending on its changing use (Fig. 3C). This toggling can be on a DHT-specific basis or a more general whole Living Lab basis. The former phenomenon is familiar in current near-linear clinical study principles for early- and late-stage DHT development (*8*, *9*, *18*).

### Living Lab platform regulatory and ethical approval

The approaches described thus far have the potential to enable flexible experimentation in the context of HCP and patient interactions in medical SaMD development (Table 1). They share one common aspect: They internalize regulatory and ethical principles as SOPs and product development processes as part of the Living Lab concept rather than trying to fight against these concepts as incompatible with and alien to a flexible experimentation space. Although the concept of uncontrolled experimentation (*28*)—where variables are observed without external manipulation—may have a role in virtual or simulated environments lacking real humans or in experimental domains that do not directly innovate or measure outcomes on living patients and HCP, it is a problematic concept in research or treatment involving human subjects. Prior international controversies and the resulting enquiries and commissions (*52*, *77*, *78*) have led to the ethical frameworks that now exist. An ethical and efficient approach for Living Labs is to enable flexibility in experimentation by designing the platform to enable efficient and near-real-time establishment and maintenance of control. Implementing a platform-based Living Lab Quality Management System locally can automate procedures and document creation using “regulation as code” methods (*79*). This approach allows for flexible experimentation while maintaining necessary control based on the risks associated with the care innovations. This requires adequate resources for staffing Living Labs with experienced quality personnel, serving as a quality unit for AI and digital innovation within the lab structure. These staff members would work alongside clinicians and researchers while maintaining independent quality responsibilities and goals focused on quality, ethical practice, regulatory documentation, and compliance (*58*). The likelihood of the approval/objection to this Living Lab approach is subject to the interpretation of individual ethics committees and competent authorities but is greater where good communication is maintained over time, and trust is built up through good Living Lab quality observation and discipline. University hospital–based Living Labs can build this trust and involve the oversight bodies in the platform. One approach could be to arrange for voluntary formal periodic audits as part of the Living Lab quality and ethics concept.

**Table 1.** Addressing the difficulty of maximizing the potential of flexible innovation offered by Living Labs while complying with rigid regulatory constraints by aligning strategies to work within the regulatory boundary.

Potential of flexible innovation by using Living Labs	Regulatory constraints that challenge the operation of flexibility of Living Labs	Solutions that align Living Lab adaptability with regulatory frameworks
Improved translation of technology from lab to real world	Living Labs are built to fit into regulatory policy that does not consider the importance of their flexible nature	Planning of the study with a wider concept of evaluation that allows taking advantage of modification of design based on feedback
Controlled real-world experimentation with advanced monitoring and data generation	Workarounds are ineffective, e.g., if a device is tested as a scientific concept for easier regulatory approval, often data cannot be used for future development	Using existing regulatory strategies developed for adaptive technologies like predetermined change control plans to allow for flexibility; ongoing/continuous ambient monitoring of data
Safe measurement and testing of technologies to analyze benefits and predict failures	Need for detailed plans, documentation, and authority approval	Taking advantage of the early TRL stage that has lower regulatory burden to evaluate scientific strategies that can later be used to inform device development
A means to bridge the gap between innovation and policy for disruptive technologies	Need for approval to make amendments in protocol that can delay processes and inhibit feedback-based development	Improved quality management systems through continuous data generation support digitization of medical devices while also complying with oversight strategies

### Expanding the Living Lab scope: Lessons from global implementations

Globally, the adoption of Living Labs across different sectors varies by country, with implementations ranging from small-scale to national-level initiatives. Regulatory support and infrastructure integration are critical for their success. Singapore offers a prime example with its recently launched Lilly’s Digital Health Innovation Hub (*80*, *81*). Aligned with the country’s National AI Strategy, the hub seeks to advance patient care and medical research by accelerating the research and development of medicinal products and medical devices including DHTs and AI-enabled DHTs. It leverages digital solutions such as passive monitoring to reduce participant burden while incorporating patient- and clinician-reported outcomes. Large datasets collected through these systems are processed using AI models to enhance quality and efficiency and drive predictive health care and personalized treatment (*80*). Another example is the Ng Teng Fong Centre for Healthcare Innovation in Singapore, a training hub for the next generation of health care professionals. It promotes team-based learning and cross-disciplinary collaboration across medical fields. Its Living Lab works closely with partners in medical technology, clinical education, and game-based health care innovations using innovative technologies to prototype and test new solutions (*82*). In South Korea, a Living Lab approach was used to design a mobile health program for Korean-Chinese women workers focused on chronic disease prevention and management. This strategy promoted active participation in every phase, from identifying needs and shaping the program structure to engaging in preevaluation (*83*). The UK also applies the Living Lab concept indirectly in patient care by using it to tackle complex health technology assessment challenges. Led by the national health technology assessment organization, NICE (National Institute for Health and Care Excellence), a collaborative forum, brings together regulators, industry, and researchers to improve decision-making in digital health through open dialogue and innovative solutions (*84*). Beyond health care, other sectors offer valuable lessons on the adoption of this concept. Multiple Living Labs exist within the EU, focusing on areas such as energy, finance, and transportation. Some have been developed with national- or EU-level support and studied in depth, offering insights for future initiatives (*29*, *85*, *86*). In Canada, the Agricultural Climate Solutions program uses a Living Lab approach to codevelop climate-resilient farming practices. Farmers and scientists collaborate on real farms to create practical solutions for reducing greenhouse gases and improving soil health (*87*). Similarly, South Korea has actively integrated the Living Lab approach into its smart city initiatives to foster citizen engagement and cocreation in urban development (*88*).

## SUMMARY AND DISCUSSION

**Fig. 4.**
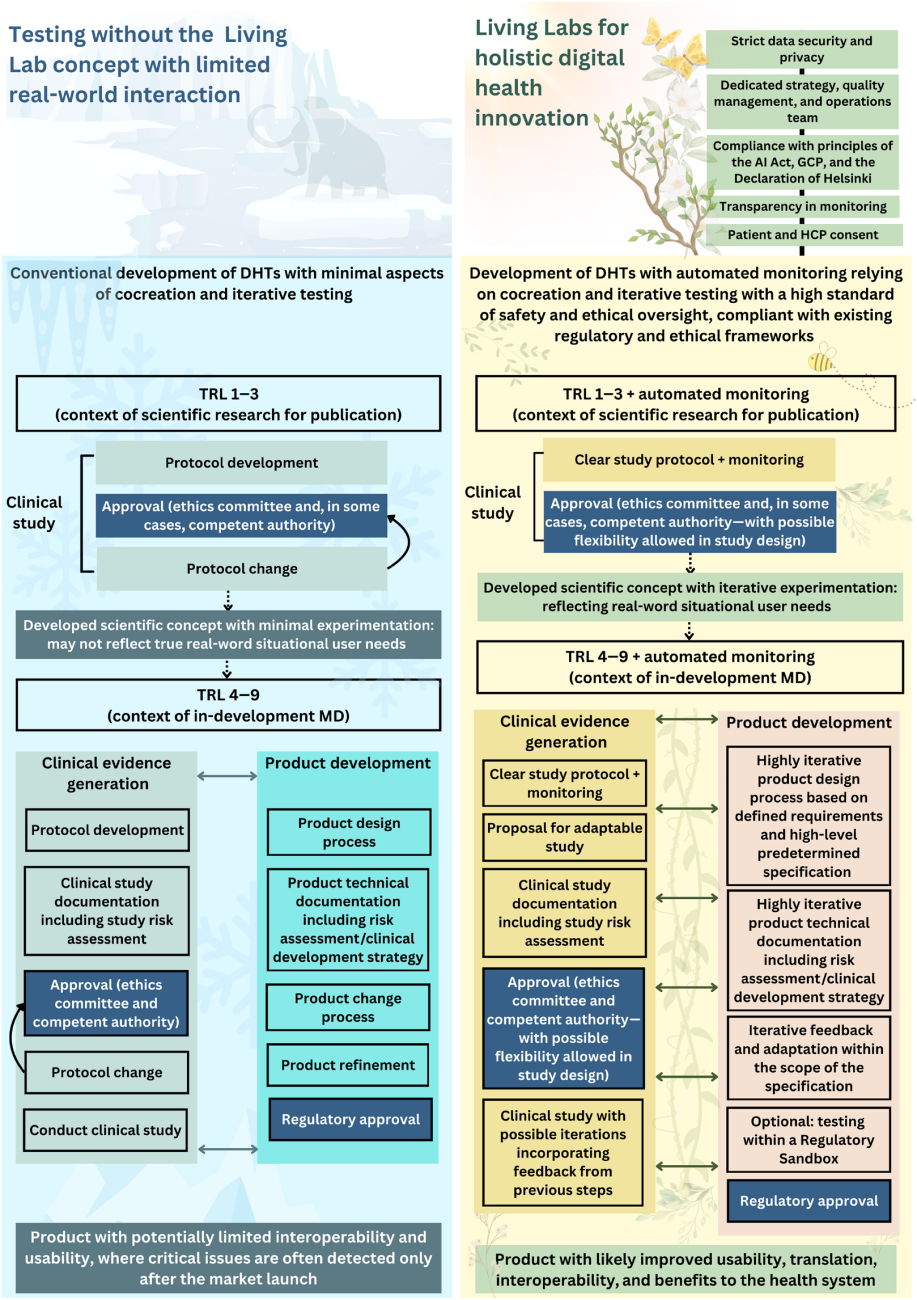
Development of DHTs using conventional strategies versus proposed Living Lab approaches. The left panel shows conventional clinical investigation strategies, which have regulatory processes with limited flexibility and evaluate products in test settings, which are often artificial or narrow. This can result in false reassurance and products that ultimately underperform in real-world use. The right panel illustrates how Living Lab strategies that involve distinct steps with stakeholders, which are aligned to TRL, can help in the development of innovative, usable products. If well planned, these approaches can comply with regulatory requirements while also better serving the needs of patients and health care professionals, enabling a smoother transition from prototype to real-world practice. This figure was created using Canva Pro.

Traditional regulatory and clinical test approaches have been developed with an understandable motivation to use measurement to achieve a definitive and binary answer to the following questions: Is this device safe, does it perform, and does it deliver a clinical benefit? As digitally enabled workflows become more complex, and solutions are increasingly interactive, changing, dynamic, and increasingly embedded into human workflows, the traditional comfortable pass/fail dichotomy easily addressed in studies that fix use and measurement increasingly becomes less relevant (*5*, *89*). Here, we propose solutions to these challenges using Living Labs that, when properly implemented, can enhance product development, improve adoption, and reduce attrition (Fig. 4). Despite their widespread application, Living Labs are not formally set out or standardized as research methodologies in health care regulatory frameworks (*48*). As the approach becomes more established, and after proving successful and providing a value in larger clinical centers, it is likely that it can be scaled to smaller centers, enabling broad adoption across diverse health care settings. However, as greater standardization is developed, it is crucial that the core concept of operational flexibility is preserved.

The EU’s vision of frameworks supporting safe innovation—including legislation such as the EU AI Act (*41*), which was the world’s first horizontal AI regulation—places complex requirements on manufacturers seeking approval for DHTs (*90*). Article 57 of the EU AI Act requires each member state to introduce at least one Regulatory Sandbox at the national level, providing controlled environments for safe AI development and testing, which must be operational by 2 August 2026. The nature of these sandboxes is set out at a high level in the AI Act, and these are relatively conservative compared to the Living Lab concept described here. They focus on mid-to-late development stages and not early development or the postmarket interactions of AI-enabled devices in real-world environments. In addition, key details on their implementation are still unclear, with potential concerns about access, backlogs, and delays (*57*, *75*, *91*). The UK has led the way with its AI-Airlock sandbox (*76*), launched by the Medicines and Healthcare products Regulatory Agency in May 2024, which began accepting manufacturer applications in September 2024, and it will explore the approval steps of two large language model–enabled medical device software programs (*92*).

For emerging AI regulations to function effectively without stifling innovation or blocking market access, Regulatory Sandboxes and Living Labs must be adequately resourced. This also applies to wider digital health medical device regulations, to the regulations applying to digital medical devices interacting with physical devices, and even to the regulation of nondigital devices, particularly those of an innovative nature. Living Labs allow for participative design and cocreation for the real-world testing of emerging technologies while ensuring that compliance remains feasible and that meaningful performance data are collected in a feasible environment for regulatory compliance alongside experimentation. Only with regulatory innovation can the AI Act function to not only ensure safe AI but to also ensure an entrepreneurial environment for AI, and this applies in the same way to all countries or federal states who will impose similar AI regulation [e.g., California (*93*)]. Setting up Living Labs requires investments and commitment from regulators to support innovative approaches. The EU has set out an ambitious regulatory vision for leading the world in the patient safety and fundamental rights oversight of AI. Such a vision can only function if an equally bold vision is set out to support innovation and entrepreneurship through the financing and supporting of innovative controlled experimentation spaces that allow AI and digital development in the real world. Without this critical investment, the practical challenges and high costs of compliance will stifle AI development in more regulated regions. A bold and well-funded strategy is required to align innovative, high-quality, and ethical digital health solutions with the core principles of medicine, ensuring safe and meaningful progress for patients and health care systems alike.

## METHODS

To support the development of this paper, we used AI-assisted tools, including ChatGPT (GPT-4 Turbo and GPT-3.5, free version, www.chatgpt.com) and ScienceOS (December, January, and February free versions, www.scienceos.ai). ChatGPT was used for refining text, improving structure and flow, and enhancing clarity, while ScienceOS assisted in expanding our literature search.

Our prompts were generally as follows:

1) ChatGPT: “Check the text for grammar and clarity.”

2) ScienceOS: “Suggest complementary citations related to (topic)”/“Is there more published literature related to (topic)?”

These tools were used to improve drafting efficiency while ensuring that all core analysis and interpretation were conducted by the authors. Figures were designed using Canva Pro (paid subscription, www.canva.com). All images were originally handcrafted by the authors, and Canva’s in-built AI image generator, “Magic Media,” was only used to refine narrow and specific image subelements.
